# Survival outcomes and healthcare utilization between immigrant patients and Danish-born patients with hematological cancers: a Danish population-based study

**DOI:** 10.1007/s10654-024-01139-z

**Published:** 2024-07-04

**Authors:** Joachim Baech, Lasse Hjort Jakobsen, Mikkel Runason Simonsen, Marianne Tang Severinsen, Henrik Frederiksen, Carsten Utoft Niemann, Peter Brown, Judit Mészáros Jørgensen, Eldad J. Dann, Søren Paaske Johnsen, Tarec Christoffer El-Galaly

**Affiliations:** 1 Department of Haematology, Clinical Cancer Research Unit, Aalborg University Hospital, Mølleparkvej 4, 9000 Aalborg, Denmark; 2 Department of Clinical Medicine, Aalborg University, Aalborg, Denmark; 3 Department of Haematology, Odense University Hospital, Odense, Denmark; 4 Odense University Hospital, Academy of Geriatric Cancer Research (AgeCare), Odense, Denmark; 5 Department of Haematology, Rigshospitalet, Copenhagen University Hospital, Copenhagen, Denmark; 6 Department of Haematology, Aarhus University Hospital, Aarhus, Denmark; 7 Department of Haematology, Rambam Medical Center, and Bruce Rappaport Faculty of Medicine, Technion University, Haifa, Israel; 8 Danish Center for Health Services Research, Department of Clinical Medicine, Aalborg University and Aalborg University Hospital, Aalborg, Denmark; 9 Department of Medicine Solna, Clinical Epidemiology Division, Karolinska Institutet, Stockholm, Sweden

**Keywords:** Hematological cancer, Immigrants, Immigration, Survival, Ethnicity

## Abstract

Overall survival (OS) for patients with a hematological cancer may differ between immigrant and Danish-born patients due to disparities in socioeconomic status, health literacy, and language proficiency. This cohort study aimed to investigate survival and hospitalization according to immigrant status while controlling for confounders. Patients with newly diagnosed hematological cancer in 2000–2020 were identified in the Danish nationwide hematological registers and stratified into Danish-born, Western, and non-Western patients. Patients were followed from diagnosis until death, 31st December 2021, or emigration, whichever came first. Crude OS, standardized OS, and 5-years OS differences were computed using flexible parametric models and hazard ratios using Cox regression. Number of hospitalization days in the year before and after diagnosis, respectively, were calculated using Poisson regression. A total of 2,241 immigrants and 41,519 Danish-born patients with a hematological cancer were included. Standardized 5-years OS was similar between groups with 58% (95% confidence interval 57–58%) for Danish-born patients, 57% (55–60%) for Western, and 56% (53–58%) for non-Western immigrant patients. Subgroup analyses identified OS differences in selected subgroups. Non-Western immigrant patients had 1.3 (0.5–2.1) more hospitalization days in the year before diagnosis and an adjusted incidence rate ratio of hospitalization days of 1.14 (1.13–1.15) in the year after diagnosis compared with Danish-born patients. In conclusion, there were no overall differences in survival when comparing immigrant patients to Danish-born patients after controlling for relevant confounders. Healthcare utilization was slightly higher among non-Western immigrant patients before and after diagnosis, but differences were small on an individual patient level.

**Supplementary Information:**

The online version contains supplementary material available at 10.1007/s10654-024-01139-z.

## Background

The Danish healthcare system provides free cancer treatments without any substantial out-of-pocket costs through tax-based funding of healthcare. However, navigating a modern, complex healthcare system is a challenge for cancer patients, many of whom are in a major life crisis. The efficiency of diagnostic work-up is influenced by the patient’s ability to arrange appointments with relevant healthcare professionals, communicate symptoms accurately, and follow complicated diagnostic pathways with several medical investigations and hospital visits. After diagnosis, patients are faced with large amounts of complex information about disease, treatment options, and prognosis, all of which forms the basis of later discussions and informed decisions regarding treatment strategies. Even for patients with full language proficiency, information received during diagnostic work-up, treatment, and follow-up are often not fully understood [1]. The consequences of poor health literacy are risk of poor treatment compliance, inability to make fully informed treatment decisions, and a delayed or no reaction to potentially life-threatening treatment complications [2]. As a result, cancer outcomes can potentially be influenced by socioeconomic status, health literacy, and general language proficiency [3, 4]. Immigrants with cancer are a potentially vulnerable patient population due to potential barriers related to language proficiency and/or cultural differences that result in healthcare-seeking behaviors and symptom communication that may be misinterpreted by healthcare professionals. For example, Danish studies have shown significant differences in healthcare-seeking behavior and outcomes among immigrant patients compared to Danish-born patients when it comes to adherence to national screening programs for colorectal-, cervical-, and breast cancer [5–7], outcomes of mental illness and stroke [8, 9], as well as diabetes-related mortality [10]. This is observed despite the fact that non-Western immigrants in Denmark have more frequent contacts to general practitioners, hospital emergency rooms, and private specialists [11–13]. Differences in healthcare-seeking behavior in immigrant patient populations has also been reported in studies from United States (US), Canada, United Kingdom, Italy, France, and Spain [14]. Many cancers are life-threatening, and survival depends on treatment availabilities. In insurance-based healthcare systems or healthcare systems with substantial co-payment requirements, immigrant patients may face additional difficulties due to financial hurdles. Therefore, a comparison of outcomes of cancers between immigrant patients versus Danish-born patients in a fully tax-financed healthcare system is of major interest, as possible outcome differences are less likely to be explainable by access differences but rather by differences in use of healthcare, communication, and healthcare-seeking behavior. This register-based nationwide cohort study aimed to compare survival outcomes and healthcare utilization between immigrant patients and Danish-born patients with a hematological cancer, and to identify patient characteristics possibly associated with survival disparities.

## Methods

### Data sources

This study was based on the Danish national health registers. Patients were identified using the nationwide quality registers for hematological cancers: The Danish Lymphoma Registry, the Danish National Acute Leukemia Registry, the Danish National Multiple Myeloma Registry, the Danish Myelodysplastic Syndrome Registry, the Danish Chronic Myeloproliferative Neoplasia Registry, and the Danish National Chronic Lymphocytic Leukemia Registry [15–21]. Merging of data from patients registered in the quality registers was possible through use of the unique civil registration number provided to all Danish citizens [22]. Dates of death were retrieved from the Danish Death Register with last follow-up data on 31st December 2021 [23].

### Study population

Patients with newly diagnosed hematological cancers (acute lymphoblastic leukemia [ALL], acute myeloid leukemia [AML], chronic lymphocytic leukemia [CLL], chronic myeloid leukemia [CML], lymphoma, myeloma, or myelodysplastic syndromes [MDS]) between 2000 and 2020 were included in the study. Patients with lymphoma were further subdivided into Hodgkin, indolent, and aggressive lymphomas. Patients with myeloma were risk stratified into low/intermediate and high risk based on the International Staging System [24]. Patients with missing information on lymphoma subtype or myeloma risk score were excluded. Immigrants and Danish-born patients were defined according to definitions used by Statistics Denmark. Immigrant patients were patients with Danish citizenship but born outside Denmark. Danish-born patients were patients born in Denmark or patients born outside Denmark but with at least one Danish-born parent with Danish citizenship [25]. Second generation immigrant patients (i.e., patients born in Denmark to parents who both are not both Danish-born and do not hold Danish citizenship) were excluded from the study.

### Patient characteristics

Information on socioeconomic factors was retrieved from the national registers: Education level was defined as the highest achieved education level at the time of diagnosis and stratified according to International Standard Classification of Education as lower or higher (Appendix 1) [26]. Income was retrieved from the Danish Salary Register and calculated as the cumulative family income in the calendar year prior to diagnosis. The calculated income levels were then grouped according to the income quartiles for Danish citizens in the same age group (five-years intervals), with first quartile being the lowest quartile [27]. The Danish Salary Register also provided information on cohabiting status. Occupational status was provided by the DREAM register and defined as the most frequently registered weekly occupational status during the 52 weeks prior to diagnosis [28]. Country of departure for immigrant patients was retrieved from the Danish Civil Registration System and classified as Western or non-Western according to definitions provided by Statistics Denmark (Appendix 2).

### Statistics

Patients were followed from diagnosis until death from any cause or censoring (31st December 2021 or emigration from Denmark), whichever came first. OS, crude and standardized for age, sex, calendar year of diagnosis, and hematological cancer subtype was computed using flexible parametric models [29]. Differences in 5-years OS were analyzed in subgroups according to patient characteristics. Hazard ratios (HR) for all-cause mortality between Western immigrant, non-Western immigrant and Danish-born patients were computed using three different Cox regression models: A crude model, a simple model adjusting for age, sex, calendar year of diagnosis, and hematological cancer subtype, and a fully adjusted model adjusting for age, sex, calendar year of diagnosis, hematological cancer subtype, cohabiting, employment status, income quartile, and education level. This was further performed in subgroups of hematological cancer subtypes. The confounders adjusted for were identified a priori in a directed acyclic graph (Supp. Figure 1). The HR from the simple and fully adjusted model, with five degrees of freedom for the baseline hazard, was plotted over calendar year comparing Western and non-Western immigrant patients to Danish-born patients. This was done by creating an interaction between immigrant status and calendar year of diagnosis, the latter modeled using a natural cubic spline with three degrees of freedom. Total number and absolute differences in number of inpatient hospitalization days during the year prior to diagnosis, and incidence rates for inpatient hospitalization days in the year after diagnosis, were calculated using Poisson regression. Crude incidence rates ratios (IRR) and IRR adjusted for sex, age, calendar year of diagnosis, and hematological cancer subtype were also reported. For the analyses of healthcare utilization, follow-up was restricted to 2018 due to data limitations, and patient inclusion years were restricted to 2018 for the analysis of hospitalization prior to diagnosis and 2017 for hospitalization after diagnosis. Data management was performed using SAS Software 9.4 [30]. Statistical analyses were performed using R version 4.0.3 [31]. The study was registered in the North Denmark Region where the study was conducted (ID: 2021–090).

## Results

### Patient characteristics

A total of 2,241 immigrant patients and 41,519 Danish-born patients with hematological cancers were included. Compared to Danish-born patients, immigrant patients were significantly younger (median age at diagnosis of 61 years vs. 69 years), had higher education level (33% classified as high compared to 22%), lower income (49% in the lowest income quartile compared to 23%), and were less likely to be retired (36% compared to 53%). Half of the immigrant patients (51%) were non-Western immigrant patients. In comparison to Western immigrant patients, non-Western immigrant patients were younger (56 vs. 65 years), with higher male predominance (59% vs. 55%), higher unemployment rate (15% vs. 5%), higher proportion in lowest income quartile (67% vs. 30%), and less likely to have a higher education (24% vs. 41%) (Table 1).

**Table 1:** Baseline characteristics for Danish-born patients, all immigrant patients, Western immigrant patients, and non-Western immigrant patients with hematological cancer

Variable	Level	Danish-born patients	All immigrant patients	Western immigrants	Non-Western immigrants
Number of patients		41,519	2241	1086	1155
Age (years)	Median (iqr)	69.0 (60.0, 77.0)	61.0 (48.0, 71.0)	65.0 (53.0, 74.0)	56.0 (45.0, 68.0)
Sex	Male	23,721 (57.1%)	1280 (57.1%)	594 (54.7%)	686 (59.4%)
	Female	17,798 (42.9%)	961 (42.9%)	492 (45.3%)	469 (40.6%)
Cohabitating	No	14,313 (36.3%)	777 (35.6%)	407 (38.4%)	370 (32.9%)
	Yes	25,122 (63.7%)	1407 (64.4%)	653 (61.6%)	754 (67.1%)
	Missing	2084	57	26	31
Employment status	Reduced work capacity	2859 (6.9%)	313 (14.0%)	73 (6.7%)	240 (20.8%)
	Employed	15,763 (38.0%)	913 (40.7%)	468 (43.1%)	445 (38.5%)
	Retired	22,066 (53.1%)	796 (35.5%)	493 (45.4%)	303 (26.2%)
	Unemployed	831 (2.0%)	219 (9.8%)	52 (4.8%)	167 (14.5%)
Income quartile	1 (lowest)	9611 (23.2%)	1084 (49.1%)	322 (30.1%)	762 (67.0%)
	2	10,546 (25.4%)	417 (18.9%)	216 (20.2%)	201 (17.7%)
	3	10,627 (25.6%)	333 (15.1%)	235 (22.0%)	98 (8.6%)
	4 (highest)	10,658 (25.7%)	374 (16.9%)	297 (27.8%)	77 (6.8%)
	Missing	77	33	16	17
Education level	Lower	31,216 (77.8%)	1357 (67.4%)	590 (58.6%)	767 (76.2%)
	Higher	8894 (22.2%)	656 (32.6%)	416 (41.4%)	240 (23.8%)
	Missing	1409	228	80	148
Time in Denmark (years)	> 10	41,519 (100.0%)	1870 (83.4%)	900 (82.9%)	970 (84.0%)
	≤ 10	0 (0.0%)	371 (16.6%)	186 (17.1%)	185 (16.0%)
Use of interpreter	Yes	19 (0.0%)	201 (9.0%)	20 (1.8%)	181 (15.7%)
Hematological cancer subtype	Aggressive lymphoma	10,658 (25.7%)	593 (26.5%)	251 (23.1%)	342 (29.6%)
	Indolent lymphoma	9432 (22.7%)	499 (22.3%)	256 (23.6%)	243 (21.0%)
	Hodgkin lymphoma	2279 (5.5%)	207 (9.2%)	104 (9.6%)	103 (8.9%)
	CLL	5611 (13.5%)	242 (10.8%)	130 (12.0%)	112 (9.7%)
	Low/Intermediate risk myeloma	3251 (7.8%)	187 (8.3%)	94 (8.7%)	93 (8.1%)
	High risk myeloma	1599 (3.9%)	79 (3.5%)	40 (3.7%)	39 (3.4%)
	ALL	415 (1.0%)	31 (1.4%)	13 (1.2%)	18 (1.6%)
	AML	4402 (10.6%)	211 (9.4%)	104 (9.6%)	107 (9.3%)
	MDS	3224 (7.8%)	125 (5.6%)	70 (6.4%)	55 (4.8%)
	CML	648 (1.6%)	67 (3.0%)	24 (2.2%)	43 (3.7%)

*ALL* Acute lymphoblastic leukemia, *AML* Acute myeloid leukemia, *CLL* Chronic lymphocytic leukemia, *CML* Chronic myeloid leukemia, *IQR* Inter quartile range, *MDS* Myelodysplastic syndrome

### Overall survival

**Fig. 1:**
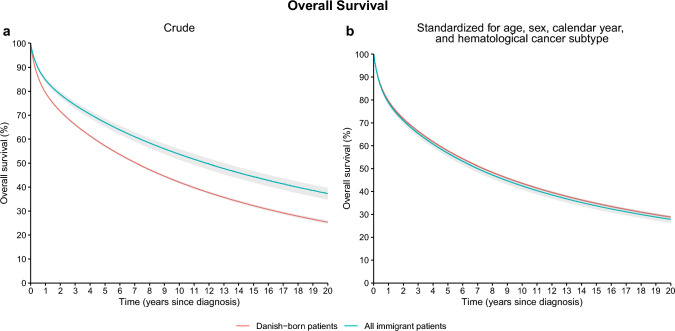
**a** Crude and **b** standardized overall survival for Danish-born patients and all immigrant patients, respectively

**Fig. 2:**
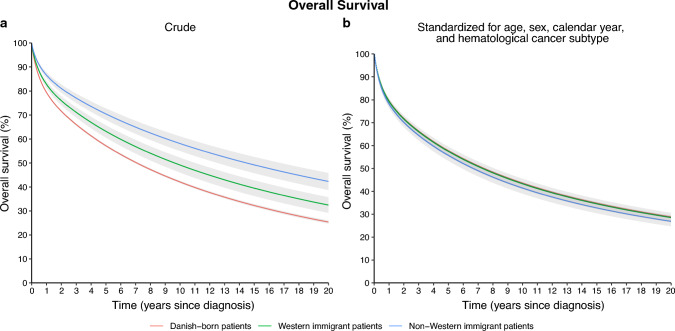
**a** Crude and **b** standardized overall survival for Danish-born patients, Western immigrant patients, and non-Western immigrant patients, respectively

Crude OS was significantly better for Immigrant patients compared to Danish-born patients (5-years OS 67% [95% confidence interval 65–69%] compared to 57% [56–57%]), but OS differences disappeared following standardization for differences in age, sex, calendar year of diagnosis, and hematological cancer subtype (5-years OS 58% [57–58%] for Danish-born and 57% [55–58%] for immigrant patients [Fig. 1]). When analyzing Western and non-Western immigrant patients separately, Western immigrants had a crude 5-years OS of 63% (61–66%) and non-Western immigrant patients had a crude 5-years OS of 70% (68–73%). When standardizing for age, sex, calendar year of diagnosis, and hematological cancer subtype, the 5-years OS was 57% (55–60%) for Western immigrants and 56% (53–58%) for non-Western immigrants (Fig. 2).

Using Danish-born patients as reference, crude HRs for all-cause mortality were also significantly lower for all immigrants, Western immigrants, and non-Western immigrants with HRs of 0.72 (0.67–0.77), 0.82 (0.75–0.90), and 0.63 (0.57–0.69), respectively. The lower risk of death was not retained after adjustments for age, sex, calendar year of diagnosis, and hematological cancer subtype with corresponding HRs of 1.05 (0.98–1.12), 1.01 (0.93–1.11), and 1.09 (0.99–1.20). Results were robust to full adjustments including age, sex, calendar year of diagnosis, hematological cancer subtype, cohabiting, employment status, income quartile, and education level with HRs of 1.02 (0.95–1.10), 1.02 (0.93–1.13), and 1.01 (0.90–1.13) for all immigrants, Western immigrants, and non-Western immigrants, respectively (Table 2). When performing fully adjusted Cox regression analyses in subgroups of each hematological cancer subtype, no statistically significant differences were found (Supplementary Table 1).

**Table 2:** Hazard ratios for all-cause mortality with 95% confidence intervals, using Danish-born patients as reference, for all immigrant patients, Western immigrant patients, and non-Western immigrant patients

Immigrant group compared to Danish-born patients	Crude HR	*P*-value	Simple adj. HR*	*P*-value	Fully adj. HR†	*P*-value
All immigrants	0.72 (0.67–0.77)	< 0.001	1.05 (0.98–1.12)	0.18	1.02 (0.95–1.10)	0.64
Western immigrants	0.82 (0.75–0.90)	< 0.001	1.01 (0.93–1.11)	0.79	1.02 (0.93–1.13)	0.62
Non-western immigrants	0.63 (0.57–0.69)	< 0.001	1.09 (0.99–1.20)	0.08	1.01 (0.90–1.13)	0.88

*Adjusted for age, sex, and calendar year of diagnosis † Adjusted for age, sex, calendar year of diagnosis, cohabiting, employment status, income quartile, and education level

*Adj*. adjusted, *HR* hazard ratio

**Fig. 3:**
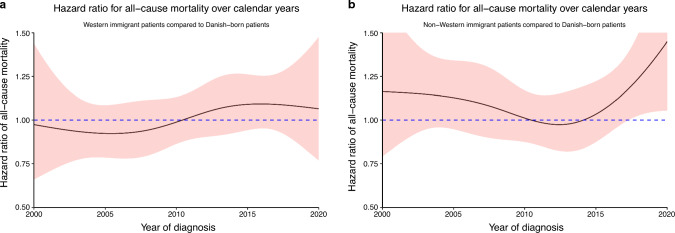
Hazard ratios over calendar year of diagnosis for all-cause mortality between **a** Western immigrant patients and **b** non-Western immigrant patients, respectively, and Danish-born patients, standardized for age, sex, and hematological cancer subtype

The adjusted HRs for all-cause mortality over calendar year of diagnosis comparing Western immigrant to Danish-born patients were not significantly different from one (meaning no difference between immigrants and Danish-born patients) during the whole period (Fig. 3A). For non-Western immigrant patients versus Danish-born patients, there was no difference in survival between 2000 and 2016, but from 2017 and onwards, there was a trend towards a higher HR for all-cause mortality for immigrants (Fig. 3B). However, after applying the fully adjusted model, there was no differences between non-Western immigrant patients and Danish-born patients for the whole period (Supplementary Fig. 2).

**Fig. 4:**
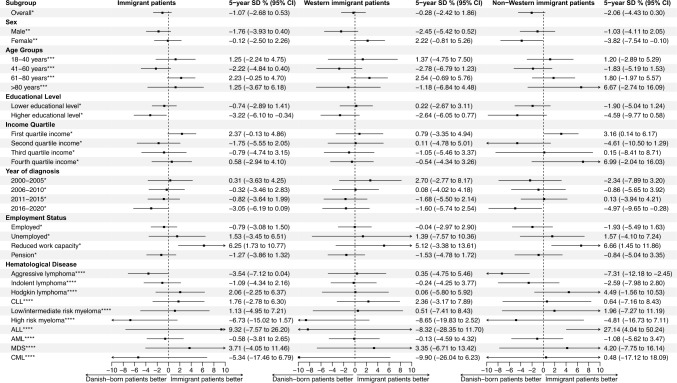
Five-years overall survival differences in percentage points with 95% confidence interval in different subgroups for all immigrant patients, Western immigrant patients, and non-Western immigrant patients, respectively, compared to Danish-born patients.* Standardized for age, sex, calendar year of diagnosis, and hematological cancer subtype.** Standardized for age, calendar year of diagnosis, and hematological cancer subtype.*** Standardized for sex, calendar year of diagnosis, and hematological cancer subtype.**** Standardized for age, sex, and calendar year of diagnosis. ALL = Acute lymphoblastic leukemia, AML = Acute myeloid leukemia, CI = Confidence interval, CLL = Chronic lymphocytic leukemia, CML = Chronic myeloid leukemia, MDS = Myelodysplastic syndrome, SD = Survival difference

5-years survival differences in subgroups were computed for all immigrants, Western immigrants and non-Western immigrants, respectively, compared to Danish-born patients. When comparing Western immigrants to Danish-born patients, there was no survival difference in any subgroups of patients. However, non-Western immigrant patients had significantly better survival when compared to Danish-born patients in subgroups of patients with lowest income quartile (3.2 percent points [0.1–6.2]), reduced work capacity (6.7 percent points [1.5–11.9]), and ALL (27.1 percent points [4.0–50.2]). Non-Western immigrants had lower 5-years OS in aggressive lymphomas (− 7.3 percent points [−12.2 – −2.5]), and patients diagnosed in the period 2016–2020 (5.0 percent points [−9.7 – −0.3]) (Fig. 4).

### Hospitalization

Prior to diagnosis, immigrant patients with a hematological cancer had a mean number of inpatient hospitalization days of 6.4 compared to 6.9 for Danish-born patients, with an adjusted difference of 0.6 days. After adjustments, Western immigrant patients had similar number of days of hospitalization compared to Danish-born patients (difference of − 0.1 days [−1.0–0.7]), but non-Western immigrant patients had an increased amount of hospitalization of 1.3 days (0.5–2.1) (Table 3).

**Table 3:** Days of inpatient hospitalization in the year before diagnosis. Difference in days using Danish-born patients as reference, for all immigrant patients, Western immigrant patients, and non-Western immigrant patients

Immigrant status	Days of hospitalization for Danish-born (mean)	Days of hospitalization for immigrants (mean)	Difference (days) (immigrants vs. Danish-born)	Adjusted difference (days) (immigrants vs. Danish-born)
All	6.91	6.42	− 0.49 (− 1.07–0.09)	0.58 (0.01–1.16)
Western		6.05	− 0.85 (− 1.67 – −0.03)	− 0.14 (− 0.95–0.66)
Non-western		6.77	− 0.13 (− 0.94–0.67)	1.30 (0.50–2.09)

In the year following diagnosis, Western immigrant patients had fewer inpatient hospitalization days compared to Danish-born patients (adjusted IRR 0.95 [0.94–0.96]), but non-Western immigrants had significantly more (adjusted IRR 1.14 [1.13–1.16]) (Table 4).

**Table 4:** Rate and incidence rate ratios (IRR) of inpatient hospitalization days in the year after diagnosis, using Danish-born patients as reference, for all immigrant patients, Western immigrant patients, and non-Western immigrant patients

Immigrant status	Rate of hospitalization for Danish-born (per year)	Rate of hospitalization for immigrants (per year)	Crude IRR (immigrants vs. Danish-born)	Adjusted IRR (immigrants vs. Danish-born)
All	30.34	32.79	1.08 (1.07–1.09)	1.05 (1.04–1.06)
Western		28.89	0.95 (0.94–0.97)	0.95 (0.94–0.96)
Non-western		36.51	1.20 (1.19–1.22)	1.14 (1.13–1.16)

## Discussion

To our knowledge, this is the first study to report OS differences between immigrant and Danish-born patients with hematological cancers. In the present study, no overall differences in OS between immigrant patients, Western as well as non-Western, and Danish-born patients were observed after controlling for confounders. Immigrants were more often in the lowest income quartile compared to Danish-born patients, but within the lowest income quartile, non-Western immigrants had superior survival compared with Danish-born patients. An explanation for this finding may be that low-income Danish-born patients were a more selected group as low-income comprised only 23% of the Danish-born patients versus 49% of immigrant patients. In the low-income group, Danish-born patients were less educated (8% classified as high vs. 18% for non-Western), more likely to be living alone (53% vs. 38%), and more often retired (55% vs. 28%) (Supplementary Table 2). Thus, Danish-born patients in the lowest income quartile appeared to have more unfavorable socioeconomic factors possibly associated with OS. Similarly, non-Western immigrants with reduced work capacity also had superior survival compared to Danish-born patients with reduced work capacity. Reasons for reduced work capacity may also differ substantially between populations.

A significant survival advantage was observed for non-Western immigrant patients with ALL compared to Danish-born patients (Fig. 4). However, in a Cox regression analysis, comparing non-Western patients to Danish-born patients with ALL using the fully adjusted model (which included socioeconomics), the survival advantage was no longer statistically significant (adjusted HR 0.33 [0.10–1.06] for all-cause mortality compared to Danish-born patients) (Supplementary Table 1). This survival advantage should be further explored, but false random associations are a risk as the ALL analyses were based on only 18 non-Western immigrants. Non-Western immigrant patients with aggressive lymphomas had a worse survival compared to Danish-born patients, which was not found for indolent or Hodgkin lymphomas (Fig. 4). After full adjustments, the HR for non-Western patients did not remain significantly elevated (HR 1.20 [0.99–1.44]) (Supplementary Table 1).

Non-Western immigrants diagnosed in the period 2016–2020 had a reduced 5-years OS compared to Danish-born patients of around 5 percent points (Fig. 4). This was also found in the simple adjusted HR for all-cause mortality plotted over calendar year of diagnosis, where there was a trend towards a higher all-cause mortality for non-Western immigrant patients compared to Danish-born patients diagnosed in the period 2017–2020 (Fig. 3B). Both results should be interpreted with caution, as the analyses are subject to uncertainty due to progressively shorter follow-up for patients diagnosed in the later part of the inclusion period. Reasons for this trend are not possible to elucidate in this register-based study. In 2018, Danish authorities cancelled financial support for interpreters in the hospital, necessitating non-Danish speaking patients, who have lived in Denmark for more than three years, to pay interpreter bills out-of-pocket. This resulted in a 33% decrease for all interpreter services in the period 2017 to 2019 in the capital region of Denmark [32]. Without interpreter support, the fraction of non-Western immigrants with poorest language proficiency may face difficulties in navigating in the complex pathways associated with diagnosis and treatment of hematological cancers. Interestingly, when the model was fully adjusted (including socioeconomic factors), OS differences were not significant at any periods (Supplementary Fig. 2).

The present study found no clinical difference in the number of days of inpatient hospitalization in the year prior to diagnosis between immigrant patients and Danish-born patients after controlling for confounders. However, non-Western immigrants had an average of 1.3 more days of hospitalization in the year prior to diagnosis, suggesting that non-Western immigrant patients required more hospital visits before being diagnosed with a hematological cancer, which could be related to language barriers preventing precise symptom reporting and efficient diagnostic work-up. Krasnik et al. reported consistent results in a study from 2002, where durations of hospital admissions were similar between immigrants and Danish-born citizens. However, Krasnik et al. did not stratify on Western and non-Western immigrant patients, and differences in hospitalization between non-Western immigrants and Danish-born citizens may have been evident upon further stratification as seen in the present study [33]. Nielsen et al. also reported highly comparable use of free-of-charge healthcare services between immigrants and Danish-born citizens [13].

In the year following diagnosis, Western immigrant patients had relative fewer inpatient hospitalization days compared to Danish-born patients (adjusted IRR 0.95 [0.94–0.96]). Western immigrants may be healthier, as shown in previous studies [34], and more resilient to treatment complications. In contrast, non-Western patients had a clinically significantly higher rate of hospitalization (adjusted IRR 1.14 [1.13–1.16]), possibly suggesting more treatment-related complications and in line with previous studies showing a higher healthcare utilization among non-Western immigrants in Denmark [11–13].

These findings are relevant beyond a Danish context, as the study explores differences by immigrant status in a healthcare setting with free access to all citizens. Therefore, patients’ ability to directly finance or co-finance cancer therapy would not have had major impact on survival outcomes, which largely eliminates the risk of poorer outcomes for immigrant patients directly caused by financial hurdles and limited access to healthcare services. In the present study, possible disparities in survival would more likely be attributed to inability to navigate/use the healthcare services offered without costs, for example because of barriers related to language proficiency, culture, or systemic inequality in the quality of care provided to immigrant patients.

Research into cancer outcomes by race, ethnicity, and immigration have generally been based on American populations and with an emphasis on racial and ethnic differences. There is a distinct difference between ethnicity, race, and immigrant status, which may translate to differences in overall survival. Ethnicity refers to the cultural characteristics of a particular group, and race is based on physical attributes, while immigrant status indicates legal residency in a new country. Individuals of different races and ethnic minorities are often born in the host country and may speak the language fluently but are still often affected by systematic discrimination, lower income, and lower education [35]. However, immigrants like those included in the present study may be more likely to be challenged by lower health literacy and language barriers in addition to discrimination. Several studies from the US have compared survival of hematological cancers for subgroups of patients based on race and ethnicity but studies are inconsistent. One study found no difference in survival for black patients with diffuse large B-cell lymphoma compared to non-Hispanic whites and another found worse 5-years OS for black patients with diffuse large B-cell lymphoma compared to non-Hispanic whites, however, both studies did not take socioeconomic factors into account [36, 37].

There have been contradicting results in the literature regarding immigrants’ health. In general, first-generation immigrants have shown better health compared to the host population in the receiving country. This phenomenon is known as the healthy immigrant effect or the healthy immigrant paradox [38]. The phenomenon is a paradox because immigrants often have lower socioeconomic position and fewer resources compared to the host population, which would otherwise be perceived as disadvantages in terms of health outcomes in general [39, 40], but also specifically in hematological cancers [41–45]. Some immigrants also originate from countries with lower life expectancy [46], but it has been shown that mortality for immigrants becomes closer to the mortality for native-born over time [47]. On the other hand, healthier individuals are more likely to emigrate than individuals with health issues, and host countries may also be more willing to accept healthy immigrants [48]. The magnitude of the healthy immigrant effect differs between European countries [49]. A systematic review from Denmark showed higher morbidity for non-Western immigrants compared to Danish-born citizens, but lower mortality [50]. The latter finding was supported by a Danish observational study of 27,134 immigrants followed from inclusion in 1993 to 1999 until 2008. The relative risks of all-cause mortality were 0.44 for women immigrants and 0.43 for male immigrants using Danish-born citizens as references and with adjustments for age and income. This supports the presence of a healthy immigrant effect in Denmark [34].

The main strength of this study is the population-based cohort from registers with almost complete follow-up. Emigration from Denmark is very limited, and all deaths are registered by the government, along with additional important information such as education level, cohabiting, occupational status, and income. In Denmark, visits to the general practitioner and the hospital are free, but co-payments are required for medications and dental care. Furthermore, treatment of hematological cancers is centralized to a few hospitals in each region of Denmark and hospital allocation is based on home region, with only very few exceptions of inter-hospital transfers. Choice or availability of hospital is therefore not dependent on living in a high- or low-income neighborhood, thus also contributing to the equalization of the impact of socioeconomic factors.

A major limitation of this study was the dichotomized stratification of immigrants into Western and non-Western immigrants as these are both very heterogeneous groups in terms of race, ethnicity, and host countries. No information on race and religion are allowed to be obtained in Danish registers and thus cannot be directly controlled for as confounders or risk factors in epidemiological studies relying solely on Danish register data. It is possible to identify information on the use of interpreters, used during hospital visits, in the Danish National Patient Register, as a pseudo marker for language barriers, but the information is not well-captured and would not contribute to the analyses performed here. Furthermore, patients with lymphoma were only stratified into aggressive, indolent, and Hodgkin lymphoma rather than specific lymphoma subtypes based on the International Classification of Diseases for Oncology, 3rd Edition (ICD-O-3). This broad stratification was chosen to provide a comprehensive overview of the survival of immigrants with hematological cancers. Further studies are needed to provide insights into the specific lymphoma subtypes, as each subtype is associated with different characteristics such as age of diagnosis, treatment intensity, and survival probabilities [51, 52]. Consequently, the impact of immigration status and socioeconomic status may affect specific lymphoma subtypes differently through various interactions, which are not captured by the current analyses.

There may be a risk of selection bias, as the study cohort in the present study only consists of patients with a hospital-diagnosed hematological cancer. There may be differences in the incidence of undiagnosed hematological cancers. If immigrants were, hypothetically, less prone to being diagnosed due to difficulties navigating the healthcare system, the present study could potentially overestimate the relative survival of immigrant patients compared to Danish-born patients. The study showed some significant differences in subgroup analyses, but multiple tests were performed, and type 1 errors cannot be excluded. Since formal adjustments for multiple testing were not performed, the subgroup analyses should only be viewed as hypothesis generating. Lastly, there were generally small numbers of immigrant patients included in these analyses, especially compared to the large number of Danish-born patients included, and the results should be interpreted with this in mind.

In conclusion, the present study found no overall differences in survival when comparing immigrant patients, both Western and non-Western, to Danish-born patients in analyses controlled for imbalances in confounders. However, the results from subgroup analyses indicate that non-Western immigrant patients with lower income and reduced work capacity had better survival than Danish-born patients. Healthcare utilization was slightly higher among non-Western immigrant patients before and after diagnosis, but differences were small on an individual patient level. While results were generally encouraging and did not reveal profound impact on survival outcomes by immigrant status, the study provides no information on quality of life, patient experience, and shared decision making, which could still be compromised in patient groups with poorer language skills.

## Electronic supplementary material

Below is the link to the electronic supplementary material.

Supplementary file1 (DOCX 369 KB)

## Data Availability

Data is not available according to rules set forth by the Danish authorities.

## Appendix 1

Education level was based on International Standard Classification of Education (ISCED) 2011 codes.

Lower: ISCED 0–4.

Higher: ISCED 5–8.

## Appendix 2

Western countries include all 28 EU countries plus Andorra, Iceland, Liechtenstein, Monaco, Norway, San Marino, Switzerland, The Vatican, Canada, USA, Australia, and New Zealand.

Non-Western countries include all other countries [53]**.**
