# Supplementary material for: Survival outcomes and healthcare utilization between immigrant patients and Danish-born patients with hematological cancers: a Danish population-based study
Source: Eur J Epidemiol. 2024 Jul 4;39(8):881–92. doi: 10.1007/s10654-024-01139-z (PMC11410925; doi:10.1007/s10654-024-01139-z)

**Supplementary Table 1.** Hazard ratios for all-cause mortality with 95% confidence intervals, using Danish-born patients as reference, for Western and non-Western immigrant patients stratified on hematological cancer subtype.

| Immigrant group compared to Danish-born patients | Crude HR | P-value | Simple adj. HR* | P-value | Fully adj. HR† | P-value |
| --- | --- | --- | --- | --- | --- | --- |
| Aggressive lymphoma |  |  |  |  |  |  |
| Western immigrants | 0.81 (0.68-0.97) | 0.02 | 0.99 (0.83-1.19) | 0.92 | 1.04 (0.86-1.25) | 0.71 |
| Non-Western immigrants | 0.73 (0.62-0.86) | <0.01 | 1.29 (1.09-1.52) | <0.01 | 1.20 (0.99-1.44) | 0.06 |
| Indolent lymphoma |  |  |  |  |  |  |
| Western immigrants | 0.84 (0.69-1.03) | 0.10 | 1.01 (0.83-1.24) | 0.90 | 0.98 (0.79-1.22) | 0.87 |
| Non-Western immigrants | 0.55 (0.42-0.71) | <0.01 | 1.14 (0.88-1.48) | 0.33 | 1.10 (0.81-1.49) | 0.56 |
| Hodgkin lymphoma |  |  |  |  |  |  |
| Western immigrants | 0.69 (0.43-1.10) | 0.12 | 1.00 (0.62-1.60) | 0.99 | 1.20 (0.74-1.96) | 0.46 |
| Non-Western immigrants | 0.40 (0.22-0.72) | <0.01 | 0.67 (0.37-1.21) | 0.18 | 0.63 (0.31-1.28) | 0.20 |
| CLL |  |  |  |  |  |  |
| Western immigrants | 0.93 (0.67-1.29) | 0.65 | 0.87 (0.63-1.21) | 0.42 | 0.79 (0.55-1.15) | 0.22 |
| Non-Western immigrants | 0.54 (0.35-0.84) | <0.01 | 0.97 (0.62-1.50) | 0.87 | 0.85 (0.49-1.48) | 0.58 |
| Low/intermediate risk myeloma |  |  |  |  |  |  |
| Western immigrants | 0.98 (0.74-1.31) | 0.90 | 0.97 (0.73-1.30) | 0.85 | 0.93 (0.69-1.25) | 0.62 |
| Non-Western immigrants | 0.63 (0.45-0.88) | <0.01 | 0.93 (0.66-1.31) | 0.68 | 0.82 (0.56-1.20) | 0.30 |
| High risk myeloma |  |  |  |  |  |  |
| Western immigrants | 1.01 (0.69-1.50) | 0.94 | 1.34 (0.90-1.97) | 0.15 | 1.40 (0.93-2.13) | 0.11 |
| Non-Western immigrants | 0.89 (0.60-1.32) | 0.55 | 1.16 (0.78-1.73) | 0.47 | 1.23 (0.76-1.98) | 0.41 |
| ALL |  |  |  |  |  |  |
| Western immigrants | 1.00 (0.47-2.13) | 0.99 | 1.36 (0.64-2.92) | 0.42 | 1.30 (0.56-2.99) | 0.54 |
| Non-Western immigrants | 0.21 (0.07-0.65) | <0.01 | 0.33 (0.11-1.05) | 0.06 | 0.33 (0.10-1.06) | 0.06 |
| AML |  |  |  |  |  |  |
| Western immigrants | 0.83 (0.67-1.03) | 0.09 | 1.01 (0.81-1.24) | 0.96 | 1.01 (0.81-1.27) | 0.90 |
| Non-Western immigrants | 0.66 (0.53-0.83) | <0.01 | 1.06 (0.85-1.33) | 0.61 | 1.06 (0.83-1.35) | 0.65 |
| MDS |  |  |  |  |  |  |
| Western immigrants | 0.85 (0.62-1.15) | 0.29 | 0.90 (0.66-1.22) | 0.51 | 0.96 (0.70-1.31) | 0.79 |
| Non-Western immigrants | 0.72 (0.50-1.03) | 0.07 | 0.88 (0.61-1.27) | 0.50 | 0.74 (0.46-1.19) | 0.21 |
| CML |  |  |  |  |  |  |
| Western immigrants | 1.21 (0.53-2.73) | 0.65 | 1.74 (0.77-3.96) | 0.18 | 1.84 (0.67-5.09) | 0.24 |
| Non-Western immigrants | 0.28 (0.09-0.88) | 0.03 | 0.93 (0.29-2.96) | 0.91 | 0.33 (0.05-2.44) | 0.28 |

* Adjusted for age, sex, and calendar year of diagnosis
† Adjusted for age, sex, calendar year of diagnosis, cohabiting, employment status, income quartile, and education level

Abbreviations: Adj. = adjusted, ALL = Acute lymphoblastic leukemia, AML = Acute myeloid leukemia, CLL = Chronic lymphocytic leukemia, CML = Chronic myeloid leukemia, HR = Hazard ratio, MDS = Myelodysplastic syndrome.

**Supplementary Table 2**. Baseline characteristics for a subgroup of Danish-born patients and non-Western immigrant patients in the lowest income quartile.

| Variable | Level | Danish-born patients | Non-Western immigrants |
| --- | --- | --- | --- |
| Number of Patients |  | 9611 | 762 |
| Educational level | Lower | 8572 (92.5) | 522 (81.6) |
|  | Higher | 692 (7.5) | 118 (18.4) |
|  | missing | 347 | 122 |
| Cohabitating | Yes | 4,288 (47.1) | 460 (62.0) |
|  | No | 4808 (52.9) | 282 (38.0) |
|  | missing | 515 | 20 |
| Employment status | Retired | 5,317 (55.3) | 216 (28.3) |
|  | Employed | 2573 (26.8) | 203 (26.6) |
|  | Reduced work capacity | 1276 (13.3) | 193 (25.3) |
|  | Unemployed | 445 (4.6) | 150 (19.7) |

**Supplementary Figure 1**. Directed acyclic graph for identification of confounders for the association between immigrant status and overall survival.


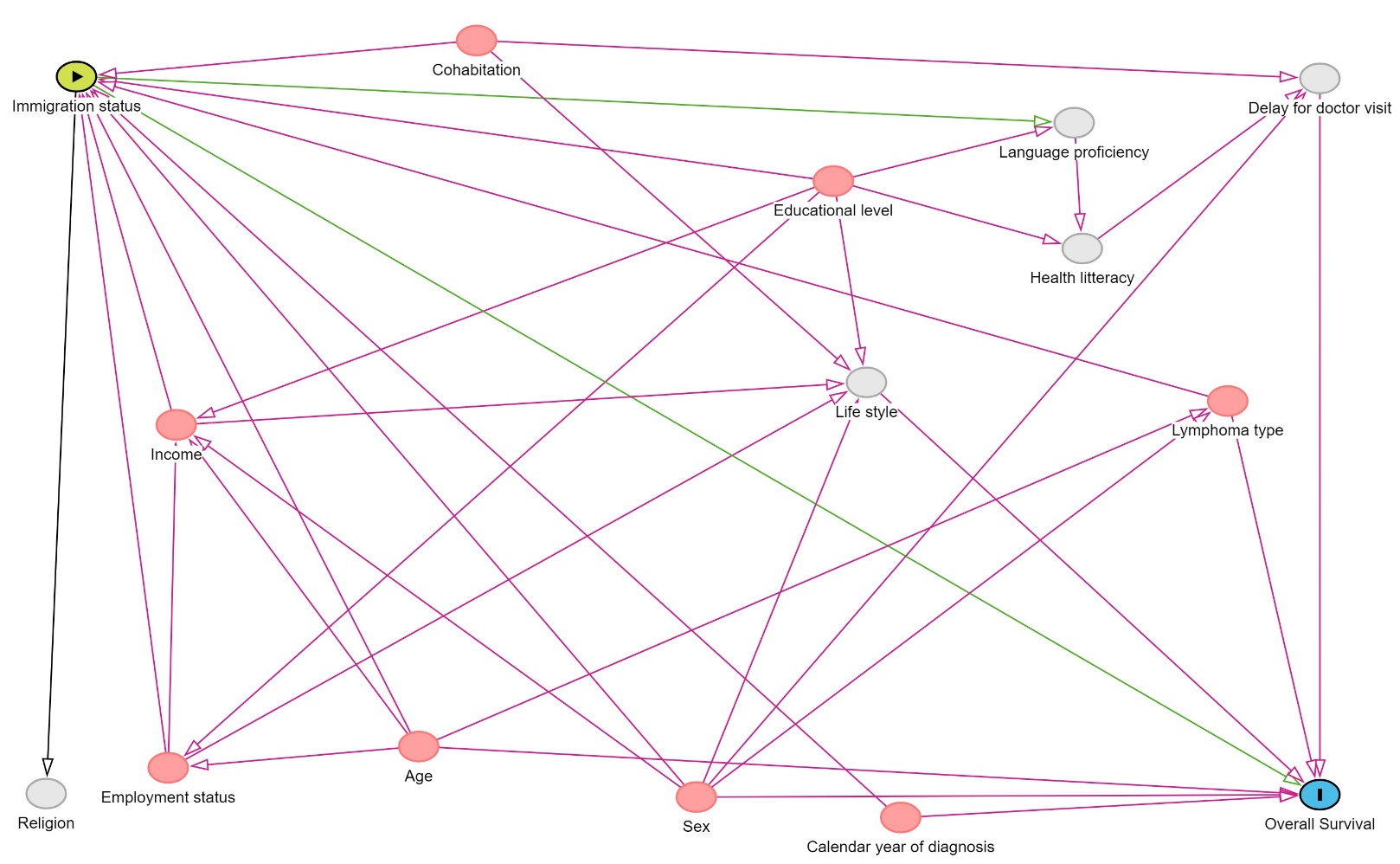


**Supplementary Figure 2**. Hazard ratios over calendar year of diagnosis for all-cause mortality between a) Western immigrant patients and b) non-Western immigrant patients, respectively, and Danish-born patients, standardized for age, sex, hematological cancer subtype, cohabiting, employment status, income quartile, and education level.
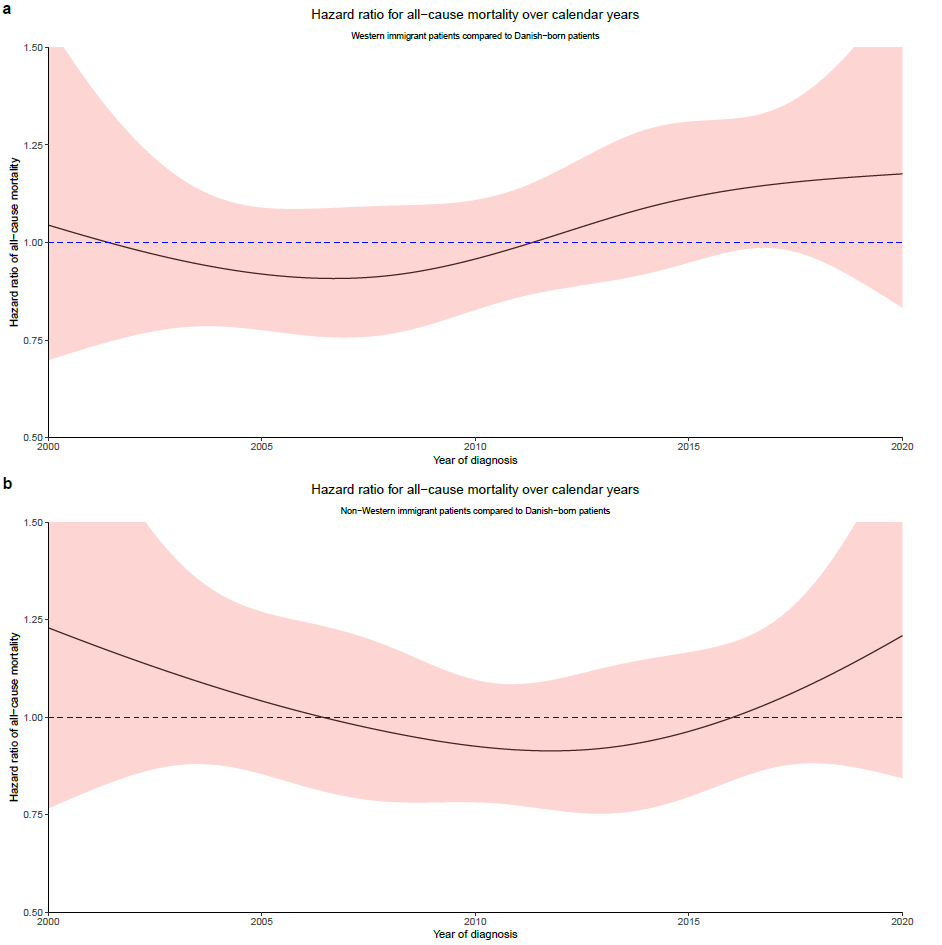

Supplement: Supplementary file 1 — Supplementary file1 (DOCX 369 KB) [file 10654_2024_1139_MOESM1_ESM.docx]
